# Parathyroid Carcinoma: Incidence, Survival Analysis, and Management: A Study from the SEER Database and Insights into Future Therapeutic Perspectives

**DOI:** 10.3390/cancers14061426

**Published:** 2022-03-10

**Authors:** Asad Ullah, Jaffar Khan, Abdul Waheed, Nitasha Sharma, Elizabeth K. Pryor, Tanner R. Stumpe, Luis Velasquez Zarate, Frederick D. Cason, Suresh Kumar, Subhasis Misra, Sravan Kavuri, Hector Mesa, Nitin Roper, Shahin Foroutan, Nabin Raj Karki, Jaydira Del Rivero, William F. Simonds, Nagla Abdel Karim

**Affiliations:** 1 Georgia Cancer Center, Medical College of Georgia, Augusta University, Augusta, GA 30912, USA; aullah@augusta.edu (A.U.); elipryor@augusta.edu (E.K.P.); tstumpe@augusta.edu (T.R.S.); luchovlz352@gmail.com (L.V.Z.); skavuri@augusta.edu (S.K.); nkarki@augusta.edu (N.R.K.); 2 Department of Pathology, Indiana University School of Medicine, Indianapolis, IN 46202, USA; khanja@iu.edu (J.K.); hmesa@iu.edu (H.M.); 3 Department of Surgery, San Joaquin General Hospital, French Camp, CA 95231, USA; awaheed@sjgh.org (A.W.); nsharma@jgh.org (N.S.); fcason@sjgh.org (F.D.C.); sforoutan@sjgh.org (S.F.); 4 National Cancer Institute, NIH, Bethesda, MD 20892, USA; suresh.kumar@nih.gov (S.K.); nitin.roper@nih.gov (N.R.); jaydira.delrivero@nih.gov (J.D.R.); 5 Department of Surgery, Brandon Regional Hospital, Brandon, FL 33511, USA; subhasis.misra@hcahealthcare.com; 6 National Institute of Diabetes and Digestive and Kidney Diseases, NIH, Bethesda, MD 20892, USA; bills@niddk.nih.gov

**Keywords:** parathyroid carcinoma, SEER program, cancer staging, incidence, survival, mortality

## Abstract

**Simple Summary:**

Parathyroid carcinoma (PC) is a rare endocrine malignancy and an uncommon cause of primary hyperparathyroidism. It is more common in older Caucasians, with a slight male predominance. Among the patients with available data in a large national cancer registry of the U.S. population, PC was usually 2–4 cm in size, histologically defined as well-differentiated adenocarcinoma, and localized to the native glands with negative lymph nodes. The overwhelming majority (>95%) of the patients underwent surgery, with the remaining few receiving radiation or chemotherapy. The 5-year survival rate after surgery was 84%. Factors such as large tumor size (>4 cm), older age (>40 years), male sex, Caucasian race, distant spread, and poor tumor differentiation were associated with an increased risk of death.

**Abstract:**

Introduction: Parathyroid carcinoma (PC) is an extremely rare entity, with a frequency of 0.005% of all malignancies. Most data related to this rare disease are limited to case series and a few database studies. We present a large database study that aims to investigate the demographic, clinical, and pathological factors, prognosis, and survival of PC. Methods: Data of parathyroid carcinoma were extracted from the Surveillance, Epidemiology, and End Results (SEER) diagnosed between 1975 and 2016. Results: PC had a slightly higher incidence in men (52.2%, *p* < 0.005), the majority of cases affected Caucasians (75.4%, *p* < 0.005), and the mean age at diagnosis was 62 years. Histologically, 99.7% were adenocarcinomas not otherwise specified (*p* < 0.005), well-differentiated (*p* < 0.005), and 2–4 cm (*p* < 0.001) in size among the patients with available data. In cases with staging provided, most PC were organ-confined (36.8%, *p* < 0.001). Lymph nodes were positive in 25.2% of cases where lymph node status was reported. The main treatment modality was surgery (97.2%), followed by radiation alone (2%), and very few received chemotherapy alone (0.8%), *p* < 0.005. Five-year follow-up was available for 82.7% of the cases. Those who underwent surgery only or radiation alone had 5-year survivals of 83.8% and 72.2%, respectively (*p* < 0.037). Multivariable analysis identified tumor size >4 cm, age > 40 years, male sex, Caucasian race, distant spread, and poorly differentiated grade as independent risk factors for mortality (*p* < 0.001). Conclusion: PC is a very rare tumor mostly affecting Caucasian individuals in the fifth decade. Older age, poor histologic differentiation, and distant metastasis are associated with a worse prognosis. Surgical resection offers the best survival outcome. To better understand the pathogenesis and factors affecting survival, all PC patients should be enrolled in national and international registries.

## 1. Introduction

Parathyroid carcinoma (PC) is an extremely rare endocrine malignancy, and the prevalence is only 0.005% of all malignancies [1,2]. The annual incidence of parathyroid carcinoma is ~3.5–5.7 cases per 10,000,000 population. PC is associated with <1% of all primary hyperparathyroidism cases. The peak incidence of PC occurs in the fifth decade of life, with no sex predilection [3]. The etiology of PC is mainly unknown; however, there is an increased risk of parathyroid carcinoma in patients with the hyperparathyroidism-jaw tumor syndrome (HPT-JT) [4]. The risk of PC may be increased in other endocrine diseases, such as familial isolated hyperparathyroidism and multiple endocrine neoplasia type 1 (MEN1) [5,6]. Several genetic mutations have been proposed in PC, including, retinoblastoma (RB), P53, cyclin D1/parathyroid adenomatosis gene 1 (PRAD1), and BRCA2; however, a strong body of evidence implicates the gene *CDC73* (formerly called *HRPT2*) first identified in the context of HPT-JT [7,8,9]. Clinical features that raise concern for parathyroid malignancy include serum calcium >14 mg/dL, PTH >5–10 times the upper limit of normal or PTH >500 pg/mL, palpable neck mass, concomitant skeletal, renal disease, and features of parathyroid crisis, e.g., altered mental status [10].

The diagnosis of PC is difficult because it overlaps clinically, radiologically, and histologically with parathyroid adenoma/hyperplasia and relies on evidence of unequivocal local tissue invasion and/or metastasis [10]. However, such evidence is often absent at the time of presentation [11]. PC is usually indolent and slowly progressive, with low rates of lymph node (<5%) and systemic metastasis (<2%) [12], and higher rates of local recurrence (25–80%). Mortality is usually from complications of hypercalcemia and not due to tumor burden [10]. Previously reported 5-year and 10-year survival rates are 76–85% and 49–77%, respectively [11]. PC is an uncommon condition with very little literature outlining its clinical aspects, diagnostic modalities, optimum treatment options, and prognostic markers [13,14,15].

We present one of the largest and most up-to-date database studies aimed at investigating the demographic, clinical, and pathological factors affecting the prognosis and survival of patients with PC.

## 2. Materials and Methods

The Surveillance, Epidemiology and End Results (SEER) database initiated by the National Cancer Institute in 1972 covers approximately 28% of the U.S. population. The SEER*Stat software (Version 8.3.5) was used to collect data using the International Classification of Diseases version 3 (ICD-O-3) and anatomical code (C75.0). There were 18 registries in all: Alaska Native Tumor Registry, Arizona Indians Tumor Registry, Cherokee Nation Tumor Registry, Connecticut tumor registry, Detroit tumor registry, Georgia Center for Cancer registry, Greater Bay Area Cancer tumor Registry, Greater California registry, Hawaii Tumor Registry, Iowa Tumor Registry, Kentucky Tumor Registry, Louisiana Tumor Registry, New Jersey Tumor Registry, Seattle-Puget Sound Tumor Registry, and Utah Tumor Registry from SEER software (https://seer.cancer.gov/seerstat/, accessed 5 March 2022). The data were exported to Statistical Product and Service Solutions (SPSS©) version 20.2 (IBM Corporation, Armonk, NY, USA).

Demographic and clinical data included age, race, sex, histologic variant, tumor differentiation, tumor size, tumor stage, lymph node status, surgical treatment, radiotherapy, chemotherapy, overall survival, survival with surgery, and survival with radiation therapy. Excluded from the final study cohort were patients with in situ malignancies, those with nonspecific tumor origins, and those without histological confirmation of cancer. This study used the “backward Wald” method to calculate odds ratios (OR) and identify the independent factors that affect survival. Data that were either unidentified or missing were removed from multivariate analysis. The chi-square test, paired t-test, and multivariate analysis were used to analyze the data. Statistical significance was defined as *p* < 0.05.

## 3. Results

In total, 609 cases of parathyroid carcinoma were identified from 1975 to 2016. 

**Figure 1:**
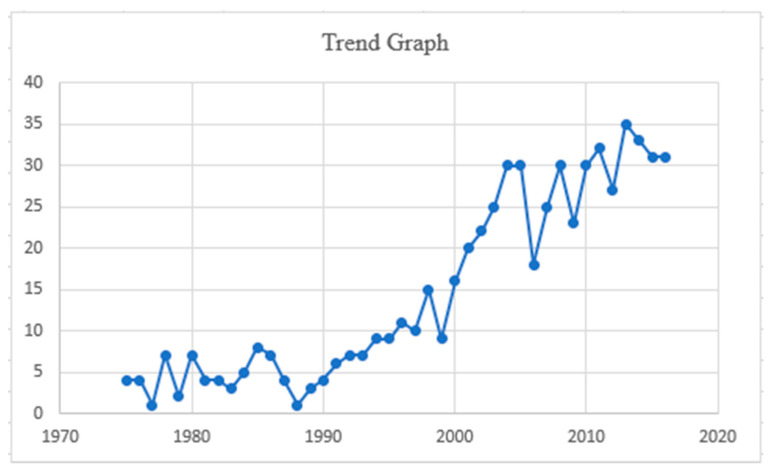
Trend analysis graph from 1975 to 2016.

Demographic data:

The mean age at diagnosis was 62, with a standard deviation (SD) of ± 10 years. Only 14% of affected individuals were younger than 40 years. There was a slightly higher incidence in men, 318 (52%), and most were Caucasians, 459 (75%). The overall cohort from 1975 to 2016 revealed an annual percentage change (APC) of 6. (Table 1 and Figure 1).

### 3.1. Tumor Characteristics and Regional Metastasis

The stage of the tumor was unknown in 267 (43.8%) cases, and the known stage was 343 (56.2%). When the tumor stage was known, 224 (36.8%) cases of parathyroid carcinoma were limited to the gland, 105 (17.2%) cases were regionally spread, and 13 (2.2%) had distant spread, *p* < 0.001. The tumor size was unknown in 388 (63.7%) and known in 221 (36.3%). Only in three (0.5%) cases of parathyroid carcinoma was the tumor size microscopic, while 58 (9.5%) had tumor size of < 2 cm, in 125 (20.5%) cases, tumor size was 2–4 cm, and, in 35 (5.7%) cases of PC, the tumor size was > 4 cm, *p* < 0.001 (Table 2).

### 3.2. Histological Types of Parathyroid Carcinoma

Histologically, 607 (99.7%) cases were adenocarcinoma not otherwise specified (NOS), and only two (0.3%) were spindle cell carcinomas, *p* < 0.005 (Table 3).

### 3.3. Histological Grading of Parathyroid Carcinoma

The histological grading of PC was unknown in 533 (87.5%), and grade was known in 76 (12.5%) cases. A total of 57 (75%) cases were well-differentiated (garde1), 13 (17%) were moderately differentiated (grade 2), 4 (5%) cases were poorly differentiated (grade 3), and only 2 (3%) PC cases were undifferentiated/anaplastic (grade 4), *p* < 0.005 (Table 4).

### 3.4. Lymph Node Status of Parathyroid Carcinoma

The lymph node status of PC cases in our study was unknown in 85 (14%) cases, and the lymph node status was known in 524 (86%) cases. The number of positive lymph nodes for PC was 153 (25.2%), and 371 (60.9%) cases in total were negative for metastatic disease spread to the lymph nodes, *p* < 0.001 (Table 5).

### 3.5. Treatment Characteristics

Most patients, 592 (97.2%), were treated surgically. Twelve (2%) received radiation alone and five (0.8%) chemotherapy, *p* < 0.005 (Table 6).

### 3.6. Outcomes and Survival Analysis

The overall 1-, 3-, and 5-year survival was 95.6%, 89.3, and 82.7%, respectively. For those treated with surgery only vs. radiation alone, the 1-, 3-, and 5-year survival was 96.6 vs. 97.6%, 90.6 vs. 81.8%, and 83.8 vs. 72.2%, respectively, *p* < 0.037 (Table 7).

### 3.7. Multivariable Analysis

Multivariable analysis identified tumor size > 4 cm, age > 40 years, male sex, Caucasian race, distant spread, and poorly differentiated grade as independent risk factors associated with increased mortality, *p* < 0.001 (Table 8).

## 4. Discussion

This study presents one of the largest cohorts of patients with PC, with mean age of diagnosis of 62 ± 10 years, slightly higher incidence in men, 25% with nodal metastasis, and tumor size 4 cm associated with poor outcome.

PC is common in the fifth decade of life, with no sex predilection [16,17]. Lee et al. [1] reported 3–19% nodal metastases and 3–4% distant metastasis at initial operation, and previous studies showed rates of less than 5% and less than 2%, respectively [10]. Reports about the impact of nodal involvement on prognosis have been contradictory: Asare et al. and other studies performed on large databases reported that survival and recurrence rates are not affected by positive nodal status [1,2,14,18]. However, single institutional studies, such as those from Harari et al. [19] and Sandelin et al. [20], reported that nodal involvement is associated with decreased survival. Lower rates of lymph node examination were reported compared to our study results [19,21]. In some multicenter studies, tumor size did not affect survival [20] and, in Asare et al., in a National Cancer Data Base (NCDB) study, tumors > 4 cm were associated with decreased survival [1,2,14,22].

Missing information from large databases and referral bias from single institutional studies may explain somewhat this discrepancy. An additional problem is that tumors with histologic features suggestive of malignancy but without evidence of invasion or metastases are classified as “atypical parathyroid adenoma/neoplasm” but may be reclassified as PC after either local recurrence or metastases have been documented, which may happen several years later [2]. A staging system for PC was recently included in the 8^th^ edition of The American Joint Committee on Cancer Staging manual; however, prognostic stage groups are not currently available due to lack of high-quality information. In this system, the extent of local invasion, but not size, is considered. For the nodal stage, only the site: central (pN1a) vs. lateral (pN1b) neck is considered [10,23].

Treatment strategies for PC mainly rely on surgical removal of the tumor [24]. In a study using data from the NCDB, Asare et al. [14] reported that complete or partial tumor resection was associated with improved survival compared with those who did not undergo surgery. A high preoperative clinical suspicion of PC should prompt the surgeon to perform a more aggressive or en bloc resection to optimize disease outcomes, since the prognosis is largely dependent on the completeness of resection and avoidance of negative complications, such as intraoperative tumor rupture [21,23,25,26].

Adjuvant therapy options for unresectable tumors include radiotherapy, chemotherapy, immunotherapy, and ablation. Management of hypercalcemia includes variable combinations of bisphosphonates, calcimimetic agents, and the osteoclast inhibitor denosumab [10,27]. Deaths due to PC are typically associated with intractable hypercalcemia rather than the effect of the tumor burden itself. Treatment of symptomatic hypercalcemia associated with PC may involve intravenous hydration, furosemide diuretics, calcitonin, mithramycin, and dialysis [22,28]. Calcimimetic agents, such as cinacalcet, which act on the calcium-sensing receptor (CaSR) responsible for regulation and hemostasis of calcium through actions on the parathyroid gland and kidney [29], are effective in reducing serum calcium levels by at least 1 mg/dl in 62% of patients with PC [9]. Intravenous bisphosphonates, including zolendronate and pamidronate, are effective in treating hypercalcemia by inhibiting the mobilization of calcium from the bone into the circulation. Zoledronate is more effective than other IV bisphosphonates in hypercalcemia associated with PC [10]. Oral bisphosphonates have not been reported to be effective for hypercalcemia associated with PC [30]. Denosumab, a monoclonal antibody targeting the receptor activator of nuclear factor kappa-b ligand, has potent antiresorptive actions in bone by blocking osteoclast formation, function, and survival [31] and has been used to treat refractory hypercalcemia associated with PC [32].

Current data on adjuvant and neoadjuvant treatments are limited. Asare et al. reported no survival benefit in 51 (7.0%) patients who received radiation treatment [14]. Some centers have reported that PC are radioresistant [23,33]; however, a retrospective review of 16 patients with parathyroid carcinoma reported 5- and 10-year survival rates of 100 vs. 80% and 69 vs. 43% for patients who received combined surgery and radiation vs. surgery alone, respectively [33]. Nevertheless, large-scale studies examining the effectiveness of combination therapy are not available.

### Molecular Insights, Ongoing Investigations, and Future Perspectives

The pathogenesis of PC likely involves the interplay of genetic and environmental factors, similar to many other malignancies. Childhood exposure to radiation, as well as concurrent thyroid or parathyroid disease increase the risk of PC [3,4]. The most consistent genetic abnormality associated with PC is mutation of *CDC73* (formerly called *HRPT2*), first identified in the context of HPT-JT [7,8,9], which encodes the protein parafibromin, a tumor suppressor gene [33,34]. The genetic syndrome most closely associated with PC is HPT-JT (at least 15% of cases). Germline *CDC73* mutations are present in up to 25% of seemingly sporadic PC cases [9]. Somatic *CDC73/HRPT2*-inactivating mutations can be demonstrated in 67% of sporadic cases [9,35,36,37]. PC has been linked to syndromes such as MEN types 1 and 2A, familial hypocalciuric hypercalcemia, and germline mutations of *CASR, RET*, and *p53* [6,10,11,14]. To date, no parathyroid cell line system has been standardized or validated, hampering preclinical research. Patient-derived tumor organoids are another preclinical model system under investigation that could greatly facilitate both basic and translational research of PC. Genetically engineered mouse models with inactivating mutations of CDC73 and/or loss of parafibromin protein expression are being investigated. Walls et al. demonstrated mice with deleted CDC73 do not develop PC [38]. However, they developed parathyroid adenoma (25%) and atypical parathyroid adenoma (75%) [38]. Amplification of *CCND1* and overexpression of cyclin D1 were observed in 41% and 82% of PC, respectively [34]. Other common somatic mutations in PC are *CDC73 (38%), TP53 (38%), MEN1 (31%), TERT (31%), PTEN* (25%), *NF1, TSC2, KDR,* and *CDK2A/B* (12% each), along with amplification of 4q12 (*PDGFRA, KIT, and KDR*) and 20q12 (*AURKA, SRC, TOP1, ZNF217, ARFRP1*) (6% each). The tumor mutation burden was low (1.7 m/Mb) except in three cases with *CDC73* mutations > 20 m/Mb [39]. The most frequent mutation was *CDC73* and TP53, which were mutually exclusive [39].

Small next-generation sequencing (NGS) studies with < 20 patients have reported actionable genetic alterations in approximately one half to two thirds of the cases [40]. Notably, NTRK1 was present in 5% and a high tumor mutational burden in approximately 20% [24,40]. The inclusion of standardized reporting systems, such as the AJCC 8th edition of the International Collaboration on Cancer Reporting (ICCR), is expected to yield useful data for establishing prognostic stages. Similarly, consistent and reliable imaging modalities for diagnosis and tumor response assessment are much needed. Fluorescent guidance has been used to identify parathyroid in thyroid surgeries to avoid hypoparathyroidism [41]. Trials testing different modes of this technology in PC to prevent incomplete resection are currently underway (Table 9). There is still an unmet need for effective therapeutic options for the management of complications, such as hypercalcemia and tumor burden. Due to the absence of consistent targetable driver mutations, use of next-generation sequencing technology will have immense value. Since MAPK, PI3K, and VEGF are overexpressed in PCs, regimens with multi-target tyrosine kinase inhibitors, such as cabozantinib, sorafenib, vandetinib + everolimus, and lenvatinib + everolimus, based on the affected pathway, have been explored with good palliative effect in a few cases [34]. A brief response to cabozantinib has been observed in patients with the *KDR T888 K* mutation, with a drop in PTH level and radiologic response [39].

Temozolomide-based regimens for high O6-methylguanine DNA methyltransferase (MGMT) promoter methylation status led to prolonged remission in a case of metastatic PC, highlighting the success of precision oncology [42]. Tumor-agnostic agents, such as pembrolizumab or nivolumab, for high tumor mutation burden or microsatellite instability-high tumors and NTRK inhibitors for PCs with NTRK mutations are applicable in selected cases. No effective adjuvant chemotherapies are currently available. Enrollment in clinical trials of novel agents should be considered for all patients when available. Genomic basket trials targeting actionable mutation(s) or immune signatures are likely the most viable options [34]. Multikinase tyrosine kinase inhibitors may be attempted on a clinical trial, with the drug either matched to the altered pathway or used empirically to block commonly altered pathways. Off-label use of the same agent as a standard of care option may be considered on a case-by-case basis. Immunotherapy against parathyroid hormone in advanced and refractory parathyroid carcinomas has shown tumor regression with hormonal and biochemical normalization [43]. Ongoing surveillance is warranted because of the risk of recurrence and distant metastasis, even after complete resection; however, surveillance intervals and modalities are not well defined. Table 9 enlists select ongoing trials enrolling patients with PCs that are registered at clinicaltrials.gov.

Limitations of our study include the limited dataset of the retrospective registry, such as incomplete data on tumor size, invasion of lymph nodes or local tissue, presence of metastasis, and specific treatment and survival outcomes. Information on the levels of calcium and PTH, as well as the effect of hypercalcemia treatment on tumor burden, survival rates, renal disease, and bone mineral density associated with PCs are not available. Furthermore, the dataset lacks information on genetic mutations, as well as association of familial cancer with PCs. An organized, national, and/or international effort is needed to gather and sequence newly identified PCs based on the criteria and protocols established collectively.

## 5. Conclusions

Parathyroid carcinoma is a very rare endocrine malignancy; the current study is one of the largest to date from the SEER database. Tumor size > 4 cm, older age, Caucasian race, male sex, poorly differentiated carcinoma, and distant metastasis were associated with poor survival rates. Surgical resection offers the best survival outcomes and is superior to radiation. Genomic profiling of tumors will help in personalized therapeutic approaches for unresectable PC. Enrollment of all patients diagnosed with PC in large-scale national and international registries will help better understand this disease.

## Figures and Tables

**Table 1:** Demographic profiles of 609 patients with parathyroid cancer from the Surveillance, Epidemiology, and End Results (SEER) database, 1975–2016.

Variable (*n* = 609)		Frequency (%)	*p*-Value
Age	20–29	24 (3.9)	<0.005
	30–39	64 (10.5)	
	40–49	100 (16.4)	
	50–59	169 (27.8)	
	60–69	132 (21.7)	
	70–79	97 (15.9)	
	80+	24 (3.9)	
Gender	Male	318 (52.2)	<0.005
	Female	291 (47.8)	
Race	Caucasian	459 (75.4)	<0.005
	African American	95 (15.6)	
	Others	55 (9)	
Trend (1975–2016 APC)	Trend	Count	
	6	609	

APC: annual percentage change.

**Table 2:** Tumor characteristics and metastasis of 609 patients with parathyroid cancer from the Surveillance, Epidemiology, and End Results (SEER) database, 1975–2016.

Variable (*n* = 609)		Frequency (%)	
Stage *	Unknown	267 (43.8)	
	Known	342 (56.2)	
	When Stage was known (*n* = 342)		*p*-value
	Localized to the gland	224 (36.8)	<0.001
	Regional spread only	105 (17.2)	
	Distant Metastasis	13 (2.2)	
Tumor Size	Unknown	388 (63.7)	
	Known	221 (36.3)	
	When tumor size was known (221)		*p*-value
	Microscopic	3 (0.5)	<0.001
	Under 2 cm	58 (9.5)	
	2 to 4 cm	125 (20.5)	
	Over 4 cm	35 (5.7)	

* = The information related to the extent of the disease (EOD) is derived from the SEER summary stage data collection system, which incorporates (EOD Primary Tumor, EOD Regional Nodes, and EOD Mets) algorithm. The EOD in this classification is assigned a code based on the extent of the disease. Various codes for EOD are used, which include in situ 0 for carcinoma in site, 1 for localized tumors to the involved organ only, 2 for direct regional extension only, 3 for the extent to the regional lymph nodes only, 4 for direct extension and regional lymph nodes involvement, 7 for distant extension, 8 for benign, borderline tumors, and 9 if extension and metastasis are unknown (https://seer.cancer.gov/archive/manuals/2021/SPCSM_2021_MainDoc.pdf, accessed 05 March 2022).

**Table 3:** Histological types of 609 patients with parathyroid cancer from the Surveillance, Epidemiology, and End Results (SEER) database, 1975–2016.

Histological Variant (*n* = 609)	Frequency (%)	*p*-Value
Adenocarcinoma NOS	607 (99.7)	<0.005
Spindle cell carcinoma	2 (0.3)	

**Table 4:** Histological grades of 609 patients with parathyroid cancer from the Surveillance, Epidemiology, and End Results (SEER) database, 1975–2016.

Grade (*n* = 609)	Frequency (%)	
Unknown	533 (87.5)	
Known	76 (12.5)	
Grade where known (*n* = 76)	Frequency	*p*-value < 0.005
Grade 1: Well-differentiated	57 (75)	
Grade 2: Moderately differentiated	13 (17)	
Grade 3: Poorly differentiated	4 (5)	
Grade 4: Undifferentiated/anaplastic	2 (3)	

**Table 5:** Regional lymph node status of 609 patients with parathyroid cancer from the Surveillance, Epidemiology, and End Results (SEER) database, 1975–2016.

**Nodal Status (*n* = 609)**	**Frequency (%)**	
Unknown	85 (13.9)	
Known	524 (86.1)	
When Nodal Status was Known (*n* = 524)		*p*-value
Negative Lymph Nodes	371 (60.9)	<0.001
Positive Lymph nodes	153 (25.2)	

**Table 6:** Treatment characteristics of 609 patients with parathyroid cancer from the Surveillance, Epidemiology, and End Results (SEER) database, 1975–2016.

Treatment (*n* = 609)	Frequency (%)	*p*-value
Surgery	592 (97.2)	<0.005
Radiation	12 (2)	
Chemotherapy	5 (0.8)	

**Table 7:** Survival data of 609 patients with parathyroid cancer from the Surveillance, Epidemiology, and End Results (SEER) database, 1975–2016.

Survival	Overall%	Surgery%	Radiation%	*p*-Value
1 year	95.6	96.6	97.6	<0.037
2 year	93.1	94	92.4	
3 year	89.3	90.6	81.8	
4 year	85.8	86.9	78.6	
5 year	82.7	83.8	72.2	

**Table 8:** Multivariate analysis of factors influencing mortality in patients with parathyroid cancer from the Surveillance, Epidemiology, and End Results (SEER) database (1975–2016).

Variables	Odds Ratio (95% C.I.)	*p*-Value
Tumor Size > 4 cm	12.1 (11.3–16.5)	<0.001
Age > 40	2.6 (2.3–2.9)	
Male gender	1.7 (1.2–1.9)	
Poorly differentiated grade	1.5 (1.3–2.1)	
Distant spread	1.9 (1.6- 2.3)	
Caucasian race	2.3 (1.9–2.7)	

**Table 9:** Selected ongoing trials in parathyroid carcinoma (source: Clinicaltrials.gov, accessed on 24 January 2022).

Trial Number	Study Title	Study Type	Intervention	Primary Outcome	Status
NCT05022641	Evaluating Impact of Near Infrared Autofluorescence (NIRAF) Detection for Identifying parathyroid Glands During Parathyroidectomy	Randomized	Intraop parathyroid eye (PTeye^®^) vs. surgeon experience during parathyroidectomy	Blood calcium levels, PTH levels	Recruiting
NCT04299425	Evaluating Impact of NIRAF Detection for Identifying Parathyroid Glands During Parathyroidectomy	Randomized	Intraop parathyroid eye (PTeye^®^) vs. surgeon’s naked eye during parathyroidectomy	Blood calcium levels, PTH levels	Recruiting
NCT02834013 (DART)	Nivolumab and Ipilimumab in Treating Patients with Rare Tumors	Non-randomized, phase 2	Nivolumab + ipilimumab vs. nivolumab	ORR	Recruiting
NCT04051099	Bilateral Superficial Cervical Plexus Block in Thyroid/Parathyroid Surgery	Randomized	Bilateral cervical plexus block vs. general anesthesia	Postop pain scores	Recruiting
NCT04344886	Optimization and Individualization of Diagnostic Scintigraphy Protocol and Minimally Invasive Radio-guided Parathyroid Surgery	Randomized	Dual-phase SPECT/CT vs. multi-phase SPECT/CT for radio-guided parathyroidectomy	Success of surgery, in-vivo and ex-vivo sensitivity, specificity and accuracy	Recruiting
NCT05152927	Near Infrared Autofluorescence (NIRAF) Detection for Identifying Parathyroid Glands During Parathyroidectomy	Randomized	Intraop parathyroid eye (PTeye^®^) vs. surgeon experience during parathyroidectomy	Number of frozen sections or parathyroid aspirate to confirm parathyroid tissue	Not recruiting

Abbreviations; intraop, intraoperative; PTH, parathyroid hormone; DART, dual anti-CTLA-4 and anti-PD-1 blockade in rare tumors; ORR, overall response rate; postop, postoperative; SPECT/CT, single-photon emission computerized tomography/computerized tomography.

## Data Availability

All data are publicly available.
